# Adherence to integrated management of childhood illness (IMCI) guidelines by community health workers in Kano State, Nigeria through use of a clinical decision support (CDS) platform

**DOI:** 10.1186/s12913-024-11245-z

**Published:** 2024-08-20

**Authors:** Megan McLaughlin, Loveth Metiboba, Aisha Giwa, Olufunke Femi-Ojo, Nirmal Ravi, Nasir Mamoud Mahmoud, Ezra Mount-Finette, Ollin Langle-Chimal, Dina Abbas, Barry Finette

**Affiliations:** 1 THINKMD, 50 Lakeside Ave, Burlington, VT 05401 USA; 2 eHealth Africa (eHA), 4-6 Independence Road, Kano, Nigeria; 3 University of Vermont Larner College of Medicine, Burlington Vermont USA, Given Medical Bldg, E-126, 89 Beaumont Ave, Burlington, VT USA; 4 Kano State Primary Healthcare Board Agency, Kano, Nigeria

**Keywords:** Integrated management of childhood illness, Community Health Worker, Child health, Clinical decision support (CDS), Digital health

## Abstract

**Background:**

The World Health Organization (WHO) Integrated Management of Childhood Illness (IMCI) guidelines established in 1992 to decrease preventable under-five child morbidity and mortality, was adopted by Nigeria in 1997. Over 20 years later, while under-five child mortality remains high, less than 25% of first level facilities have trained 60% of community health workers (CHW) who care for sick children with IMCI. This study investigated the impact in CHWs overall adherence to IMCI guidelines, particularly for critical danger signs, as well as usability and feasible following the implementation of THINKMD’s IMCI-based digital clinical decision support (CDS) platform.

**Methods:**

Adherence to IMCI guidelines was assessed by observational and digital data acquisition of key IMCI clinical data points by 28 CHWs, prior, during, and post CDS platform implementation. Change in IMCI adherence was determined for individual CHW and for the cohort by analyzing the number of IMCI data points acquired by each CHW per clinical evaluation. Consistency of adherence was also calculated by averaging the percentage of total evaluations each data point was observed. Usability and acceptability surveys were administered following use of the CDS platform.

**Results:**

THINKMD CDS platform implementation notably enhanced the CHWs’ ability to capture key IMCI clinical data elements. We observed a significant increase in the mean percentage of data points captured between the baseline period and during the CDS technology implementation (T-test, t = -31.399, *p* < 0.016, Holm-Bonferroni correction, two-sided), with the mean values going from 30.7% to 72.4%. Notably, even after the completion of the technology implementation phase, the mean percentage of IMCI elements captured by CHWs remained significantly elevated compared to the baseline, with a 26.72 percentage point increase (from 30.7% to 57.4%, T-test, t = -15.779, *p* < 0.05, Holm-Bonferroni correction, two-sided). Usability and feasibility of the platform was high. CHWs reported that the CDS platform was easy to learn and use (93%) and enabled them to identify sick children (100%).

**Conclusion:**

These results demonstrate that utilization of a digital clinical decision support tool such as THINKMD’s IMCI based CDS platform can significantly increase CHW adherence to IMCI guidelines over paper-based utilization, increase clinical quality and capacity, and improve identification of key danger signs for under-five children while being highly accepted and adopted.

**Supplementary Information:**

The online version contains supplementary material available at 10.1186/s12913-024-11245-z.

## Background

In 2019, the global under-5 mortality rate (U5MR) was 37·7 deaths per 1000 live births, with an estimated 5·2 million deaths, a majority of which could have been prevented with accurate clinical assessments and appropriate delivery of basic primary healthcare. Nearly half of these deaths (49%) occurred in 5 countries: Nigeria, India, Pakistan, Democratic Republic of the Congo, and Ethiopia. Across Nigeria the U5MR was 117·2 deaths per 1000 live births, a statistic that was significantly higher in the Kano State region, where U5MR was 203 per 1000 live births [1, 2]. Keeping with these global trends, an estimated 48·1 million under-5 deaths are projected to occur between 2020 and 2030, of which almost 23% (11 million) are preventable [1].

The leading cause of under-5 deaths are neonatal disorders followed by respiratory infections, diarrheal diseases, congenital birth defects, and malaria [3]. In 1995, the World Health Organization (WHO) and UNICEF (United Nations Children's Fund) developed Integrated Management of Childhood Illness (IMCI), a comprehensive clinical strategy that focuses on addressing the top causes of morbidity and mortality in under-5 s in low and middle-income countries (LMIC). Moving away from a single clinical diseases or conditions approach, IMCI uses an integrated approach comprising four components: 1) improving case management skills of all health-care staff 2) improving overall health systems, 3) improving clinical healthcare capacity and 4) improving family and community health practices.

IMCI has been shown to improve health worker practices and quality of care when appropriately implemented [4]. A 2016 Cochrane review found that effective implementation of IMCI reduced child mortality by up to 15% [5, 6]. Despite the benefits, comprehensive IMCI programming has been slow to scale and challenging to implement. As of 2017, only 44 of 94 countries had implemented all three IMCI components in more than 90% of districts [7]. Lack of political support, limited human and material resources, the time commitment required of health workers, including the time and expense taken for complex training that results in absence from other duties, and inadequate funding for the required training are often cited as challenges to being able to scale and operationalize IMCI globally [8, 9].

Nigeria, which adopted IMCI in 1997, faces similar barriers to implementation. National uptake remains low, with less than 25% of first-level facilities having trained staff on IMCI. Kano state, one of the first six states where the IMCI program was rolled out, has experienced poor adherence and compliance to IMCI protocols, primarily because of inadequate training and monitoring [10]. The consequence of such poor training is continuation of poor quality of care, leading to low utilization of services. A study conducted on awareness and implementation of IMCI among pediatric nurses in Southwest Nigeria revealed that while over 50% of the respondents had fair knowledge and implementation of IMCI completion and proper use of the guidelines was limited [11]. Nurses cited lack of training and the time required for implementation as primary challenges. In the absence of professional implementation aids (walls charts, booklets), nurses had to improvise when assessing and treating patients.

To circumvent the training challenges, countries have shortened the original IMCI training [12], which is designed as an 11-day in-service course that combines theoretical and hands-on clinical sessions [13]. Standard implementation is guided by an IMCI chart booklet, a large paper register that prompts health workers to follow a step-by-step algorithm to help diagnose, triage, and treat illness. The booklet also serves the dual purpose of capturing data that can be used for the monitoring and supervision of IMCI programs. Although the booklets are simple, following this process for each case can be labor and time intensive and serves as a deterrent to proper compliance and quality of care.

In this study, we assessed whether the introduction of THINKMD—an IMCI compliant digital CDS platform—can lead to meaningful improvement to adherence to IMCI guidelines and acquisition of data points that identify key danger signs. The study also evaluated the acceptability and usability of the platform among community health workers (CHWs) performing clinical risk assessments of children 2 – 59 months of age. The study also documented whether introduction of a digital platform can help ease the burden of training, monitoring, and supervision that are necessary for IMCI adoption, sustainability, and health outcome impact. Other pilot projects have reported on the importance and challenges associated with introducing digital technologies to assist with primary health care implementation [14].

The digital CDS platform used in this study was developed by THINKMD [15]. The platform is a clinical decision support platform that guides users through a comprehensive assessment in which key IMCI based clinical data elements are acquired and then analyzed utilizing physician-based Bayesian logic to generate integrated clinical risk and severity assessments, triage recommendations, treatment options [16]. The platform interprets 42 key clinical data points based on WHO IMCI and Integrated Community Case Management (ICCM) guidelines and protocols, as well as other evidence-based data points that allow for additional evaluations. Initial clinical validation studies of this platform demonstrated an 85–95% correlation between integrated clinical assessments and triage recommendations generated by CHWs to that of local physicians independently evaluating the same child [15].

## Methods

### Study design

**Fig. 1:**
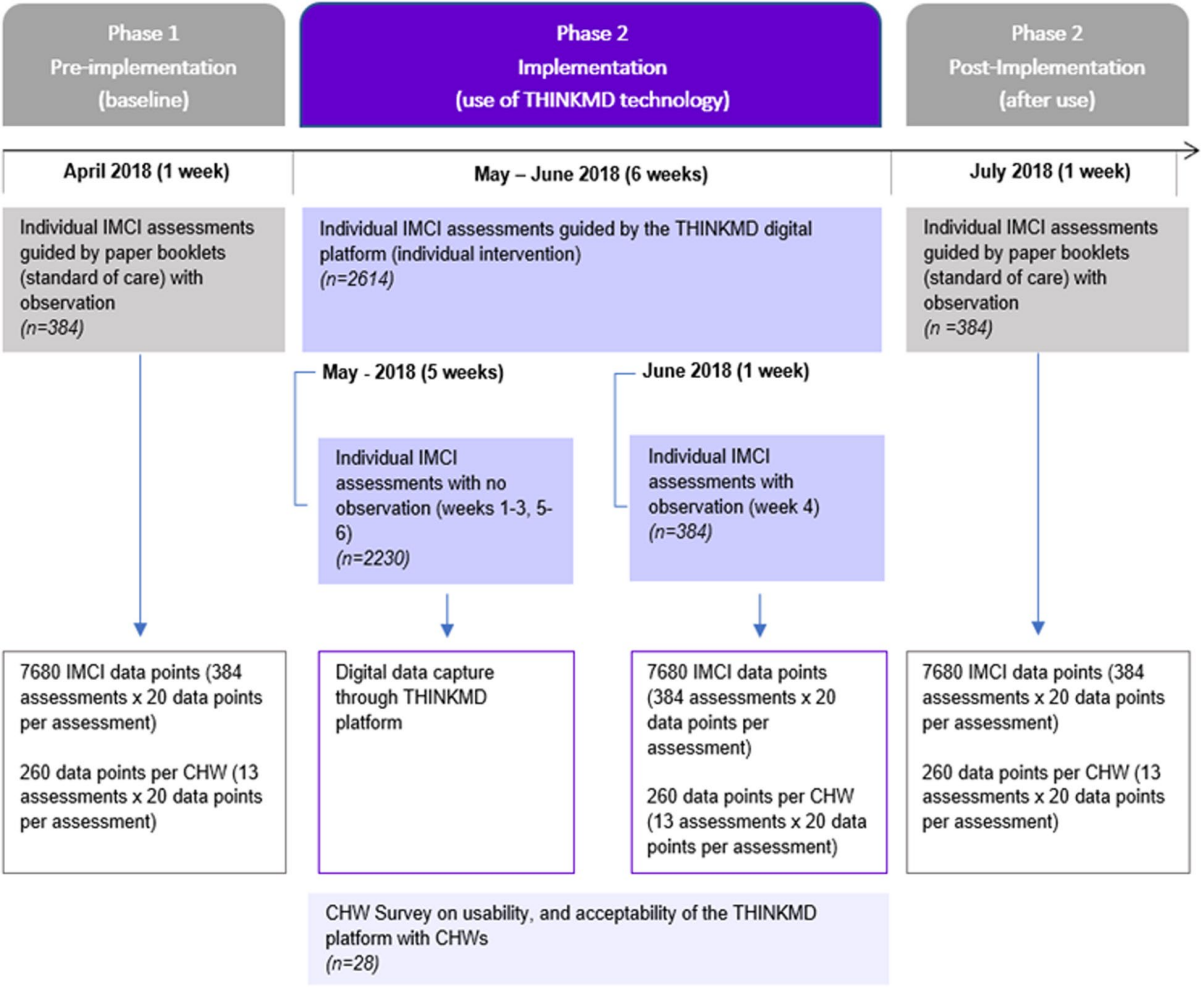
Study flow

The quasi-experimental study occurred in 3 Phases: Phase 1 (baseline pre-technology implementation); Phase 2 (CDS technology implementation); Phase 3 (post-CDS technology implementation). At each phase we observed CHW’s ability to capture up to 20 key IMCI clinical data elements for each patient evaluation over a week period. During Phase 1 and Phase 3, CHWs used IMCI paper booklets, which is the standard practice of care in this setting. During Phase 2, CHWs were trained and performed patient evaluations using the CDS platform. During Phase 2, IMCI data point acquisition was captured via both observed as well as digitally captured clinical IMCI data points via the CDS platform. The study flow is shown in Fig. 1.

**Fig. 2:**
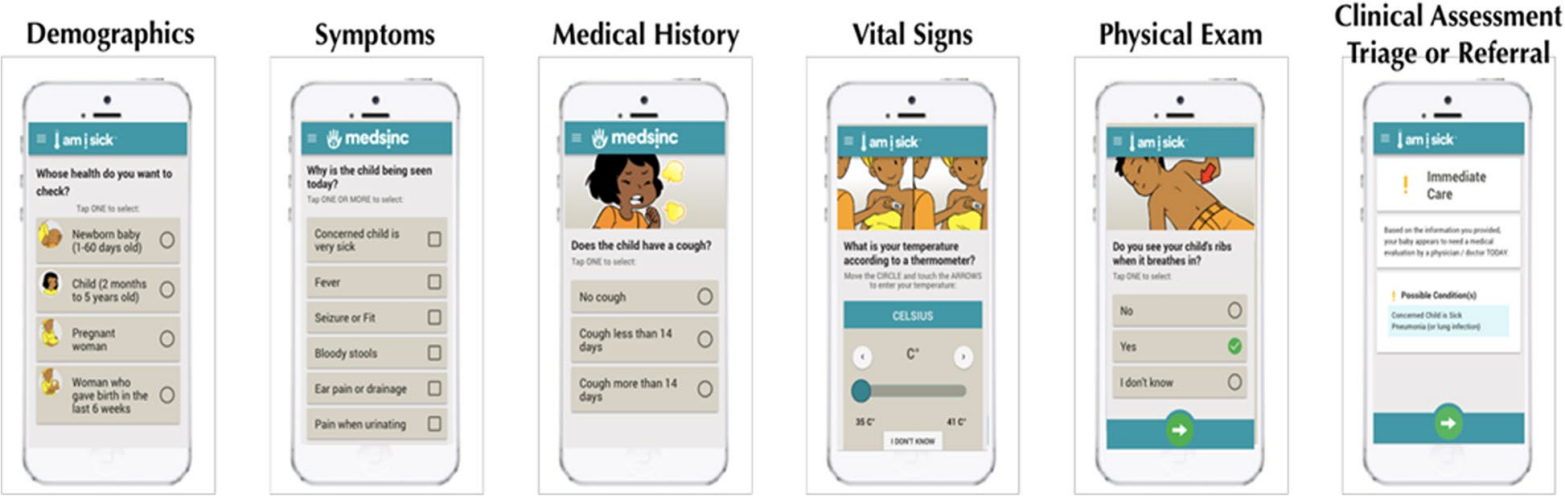
Clinical decision support platform panels

To complete an evaluation on the CDS platform, the CHWs were required to enter data for 32 panels: 11 documenting health history, six based on observation of the patient, and 15 that require physical assessment (Fig. 2). These 32 data points, along with additional metadata on user interaction, geolocation, site location and device use were also acquired.

### Procedures

**Fig. 3:**
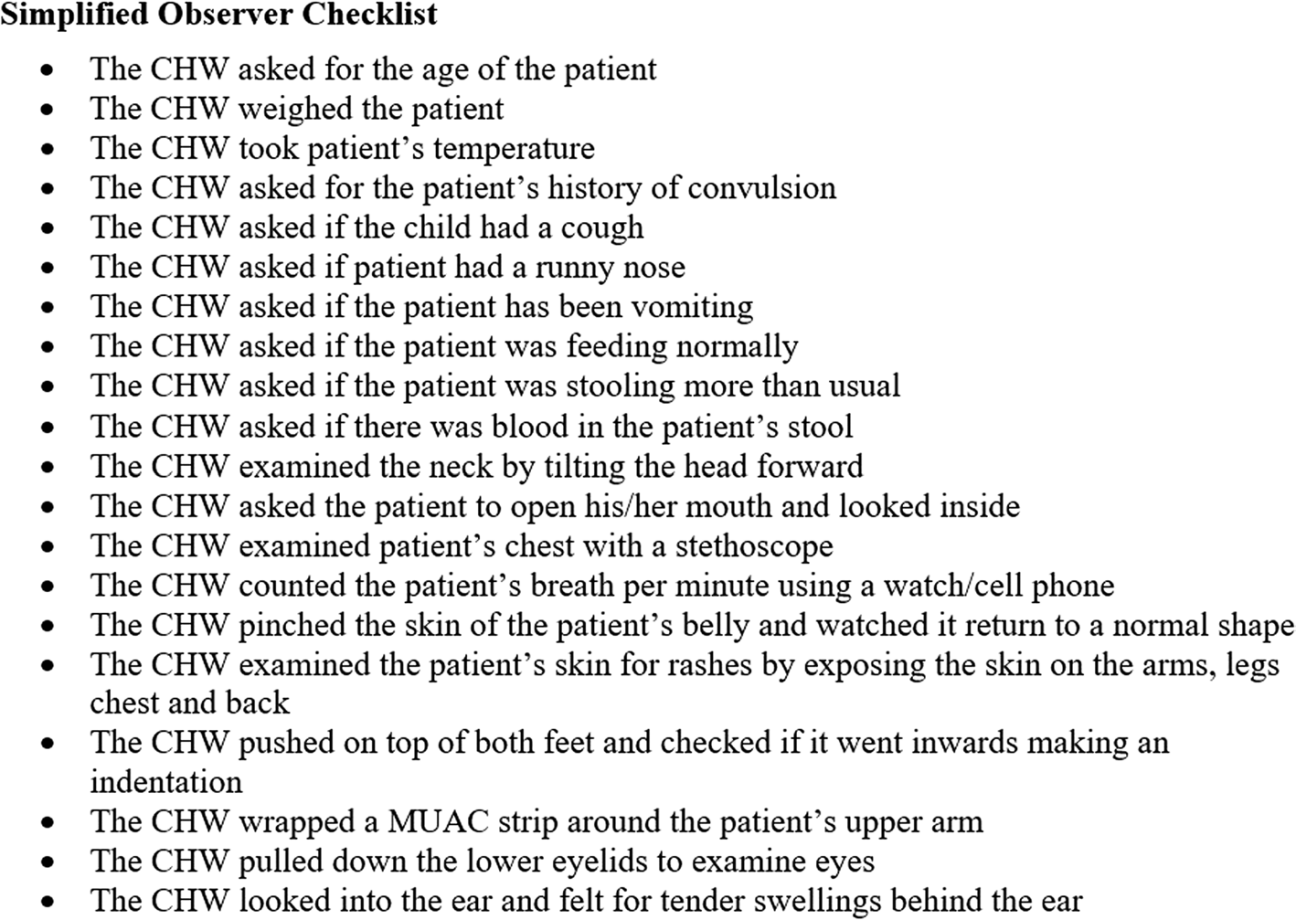
Simplified observer’s checklist

Throughout the three study phases, study observers documented CHWs performing IMCI evaluations of children aged two to 59 months. Study observers used a simplified observational checklist (Fig. 3) developed for this study to assess CHW’s adherence to, and compliance with, IMCI protocols (Supplementary file 1). The checklist was adapted from the Nigerian Federal Ministry of Health IMCI training manual and designed for the study. It captured an abbreviated list of 20 IMCI key data points that had been selected based on importance and ease of observational acquisition.

The total time allocated for community-based study was eight weeks, of which one week was allocated to data collection for phases one and three. Phase two occurred over six weeks during which study observers observed CHWs for one week (week 4) to capture CHW’s observational adherence to IMCI protocol using the simplified observational checklist. Throughout the six weeks in Phase 2, the CDS platform digitally captured information on 32 IMCI data points. At the conclusion of this phase, CHWs were surveyed about their usability and acceptability experience with the CDS platform.

Prior to study launch, the study protocol was reviewed and approved by University of Vermont and Nigerian Ministry of Health Institutional Review board and Committee for Human Research, Burlington Vermont, USA, and Kano State, Nigeria. As approved by both ethical committees, verbal/oral informed consent was obtained from either the caregivers or legal guardian, and from children if they were able to assent. In accordance with institutional requirements, written informed consent from the participants' legal guardian was not required. All children assessed during this study were provided appropriate care and referrals by a designated frontline health professional.

This study was conducted across five Local Government Areas (LGAs): the Kano Municipal Council, Tarauni, Nasarawa, Gwale and Dala in Kano State, Nigeria. A non-probability sampling method was used to identify 30 CHWs associated with the general out-patient department (GOPD) and maternal and child health (MCH) Departments in Primary Health Center (PHC) facilities in this catchment area. In each phase, CHWs were assigned to conduct a maximum of 13 IMCI evaluations of children aged two months to five years. Ten observers were selected from 16 health facilities (10 PHC, 4 Health Clinics, 2 Health Posts) in the same LGAs. The selection criteria for observers were fluency in Hausa language, prior experience conducting clinical and child health related research for other eHealth Africa partners and collaborators, and prior experience as a CHW. Observers were assigned to between two to three CHWs, whom they followed throughout the study.

All study observers were required to attend a half day training on study procedures and data collection methods. The training curriculum included case-based practical sessions to practice the simplified observer’s checklist. At the start of Phase 2, study CHWs were trained in two separate one-day sessions. In the first session, THINKMD employed a distance-learning method to train eHealth Africa staff (as master trainers), along with 15 study CHWs through a remote video platform (Zoom). This session focused on a Train the Trainer strategy and included topics on use of a digital tablet, use of the CDS platform, and technical training on IMCI physical assessment and observations. Practice sessions reviewed vital sign acquisition using a metronome, case study testing and role play. eHealth Africa staff led the second training session, covering the same topics with the other 14 study CHWs.

### Statistical analysis

A comparative analysis of the observed data points analyzed three outcomes across the three phases of the study: adherence to the IMCI protocol and consistency in implementing the protocol. CHW adherence was defined as how complete the assessment was as associated with each of the 20 IMCI data points included in the study checklist. Consistency was determined by how frequently CHW completed their assessments (e.g. maintained adherence) across each of their assigned evaluations. We determined both individual compliance during each phase as well as compared mean IMCI compliance at baseline pre-technology implementation, during technology implementation, and following the post-technology implementation. The total data points acquired during each phase of the study was 7680 (384 total CHW evaluations × 20 IMCI data points per evaluation).

## Results

This study was performed over an eight-week period between April and August 2018. The study occurred in 3 Phases: Phase 1 (baseline pre-technology implementation); Phase 2 (technology implementation); and Phase 3 (post-technology implementation). A total of 384 CHW evaluations (7680 IMCI observation data points) were performed during the two later phases of the study by 28 CHWs. During Phase 1 there were 30 CHWs but after it 2 were removed from the pool. For consistency, we dropped the evaluations of those two CHWs from the baseline period, leaving us with a total of 358 evaluations (7160 IMCI observation data points).

During Phase 2, CHWs performed a total of 2614 individual evaluations over a six-week period. During this implementation phase, IMCI data points were digitally captured via the CDS platform.

### Adherence to IMCI Protocol

CHWs were tasked with completing 13 individual evaluations in each phase and adherence was evaluated by using the study checklist to document how many of the 20 key IMCI data points were completed for each evaluation. The total number of datapoints that could be completed by any CHW in each phase was 260 (13 evaluation × 20 IMCI data points per evaluation). In Phase 1, on average, CHWs completed 30.7% (*n* = 60) [Range: 13% (*n* = 34/260) – 77% (*n* = 199/260)] of the 20 IMCI data points possible during the evaluation. This observed average increased to 72.4% (n = 169) in Phase 2 (when using the CDS platform). An overall 41.7% increase in observed adherence to IMCI guidelines. Twenty-six 26 of the 28 CHWs completed more IMCI data points in Phase 2, whereas there was a decrease in the number of datapoints completed for two CHWs.

**Fig. 4:**
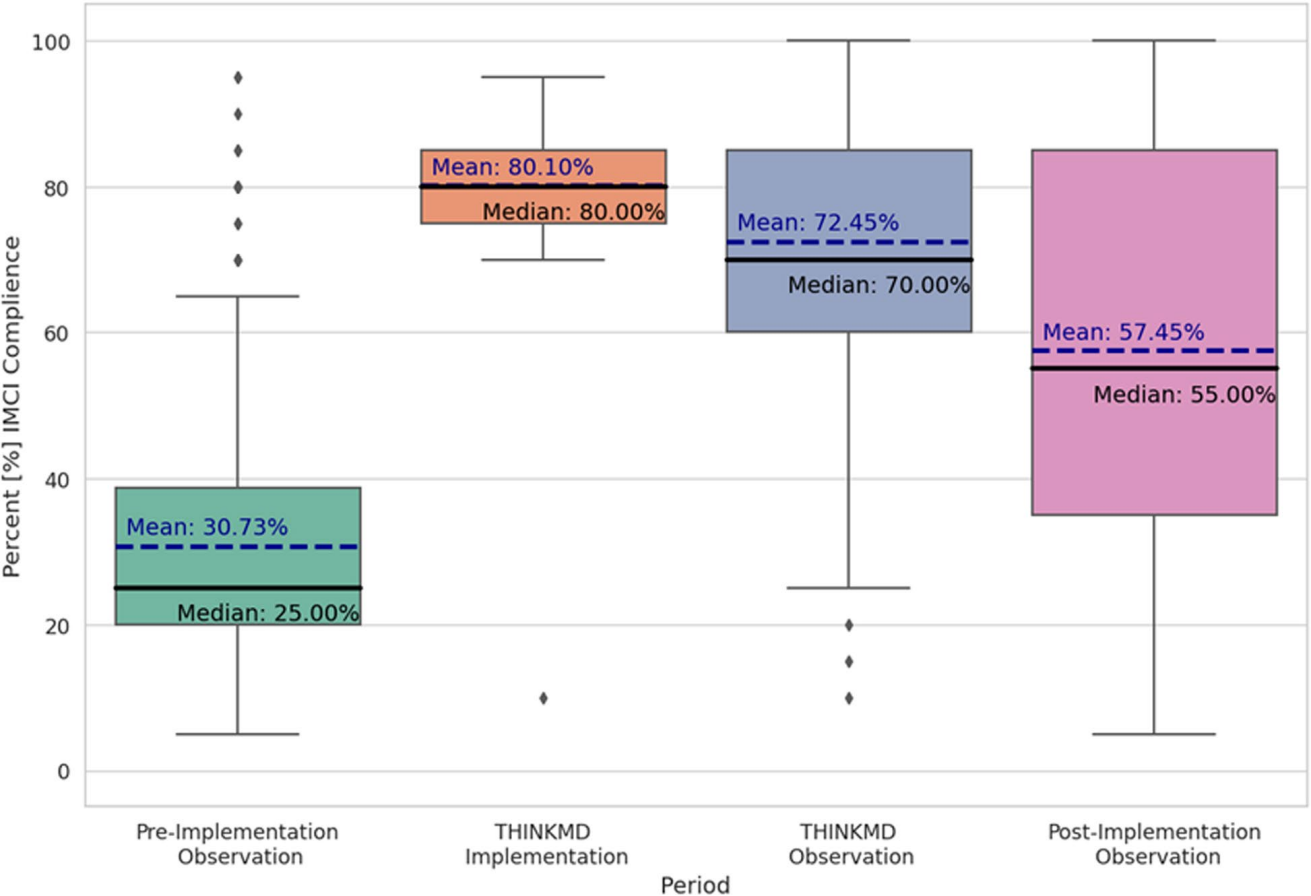
IMCI Compliance (%) Pre, During, and Post CDS Implementation. This figure shows the percent IMCI compliance at each study phase (pre, during, post) using both observed compliance (observer’s checklist) and actual compliance (THINKMD data input)

Compared to Phase 2, CHWs completed fewer data points (57.5% (*n* = 153/266) in Phase 3 (Table 1). However, compared to Phase 1, there was an overall 26.7% increase in observed adherence to IMCI guidelines across the CHW cohort in Phase 3, with overall 93% (*n* = 26 / 28) CHWs demonstrating improved clinical diagnosis skills. Summary of this data with respect to IMCI compliance is shown in Fig. 4.

**Table 1:** Rates of observed adherence and consistency with IMCI protocols across the three study phases

	Phase 1 – Baseline /pre-implementation *n* = 358	Phase 2 – Implementation *n* = 384 (week 4)	Phase 3 – post-implementation *n* = 384
**Adherence with IMCI protocol (Observed)**	**30.7% (88)**	**72.4% (250)**	**57.4% (227)**
Assessed cough	55.3% (198)	93.2% (358)	80.7% (310)
Counted breaths	5.5% (20)	80.7% (310)	45.5% (175)
Inquire about stool frequency	49.1% (176)	93.4% (359)	80.2% (308)
Took temperature	67.0% (240)	72.65% (279)	74.7% (287)
**Consistency with IMCI protocol (Observed)**			
Capture convulsion	12.8% (46)	30.7% (118)	33.0% (127)
Captured vomiting	50.5% (181)	94.5% (363)	78.1% (300)
Captured feeding	48.8% (175)	89.5% (344)	72.9% (280)
Key indicators of dehydration—Skin turgor	9.49% (34)	83.5% (321)	47.3% (182)
Key indicators of malnutrition—Pitting edema	7.5% (27)	83.8% (322)	40.3% (155)
Key indicators of respiratory distress – breathing rate	5.5% (20)	80.7% (310)	45.5% (175)

### Consistency with IMCI protocol

When comparing how consistently CHWs completed each IMCI data point across IMCI evaluations from Phase 1 to Phase 2, the study found an average percentage point increase of 5–76% in the number of assessments each data point was observed by the CHW cohort. CHWs captured danger signs (convulsion, vomiting, and feeding) significantly more (17.9%, 44%, 40.7%, respectively) when using the CDS platform (Phase 2) as compared to when they relied on the IMCI paper booklets during Phase 1 (Table 1).

**Fig. 5:**
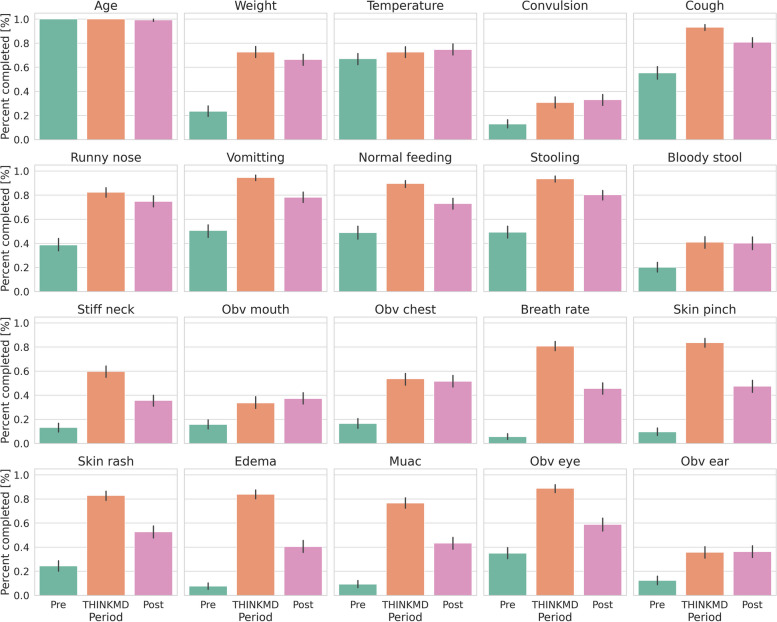
Calculated standard deviations for IMCI data point capture by phase of study. This figure shows the percentage acquisition of key clinical data points during a health assessment at each phase of the study (pre, during, and post use of THINKMD)

During Phase 1, CHWs also rarely assessed skin turgor [9.4% (*n* = 34)], Pitting edema [7.5% (*n* = 27)], and breathing rate [9.4% (*n* = 20)], the key indicators of dehydration, malnutrition, and respiratory distress, respectively. Acquisition of these data points increased with use of the CDS platform to 83.5% (*n* = 321) and 83.8% (*n* = 322) both skin turgor and pitting edema, respectively, and to 80.7% (*n* = 310) for respiratory distress when documented by study observers using the simplified checklist and to 100%, as captured by the digital platform. A smaller increase in capture of danger signs and key indicators was sustained in Phase 3, after CHWs returned to using paper booklets (Table 1). In general, we found that all IMCI data points but age and temperature (T-test, *p* > 0.02, Holm-Bonferroni correction, two-sided) measurements had a significant increase in their collection between baseline and Phase 2 and 3 (Fig. 5).

### Usability and acceptability of the CDS platform

The 28 CHWs who participated in Phase 2 technology implementation were surveyed following the completion of the study to assess overall usability and acceptability of the CDS platform. CHWs found the CDS platform easy to use with 89% (*n* = 25) stating that it was easy or very easy to learn how to use; 93% (*n* = 26) stating that it was easy or very easy to use on a five-point Likert scale (1 = very difficult, 5 = very easy). CHWs said that the CDS platform allowed them to do their job better and that it was useful in assisting with clinical assessments and diagnosing sick children. CHWs (82%) said that they were likely or extremely likely that they would recommend the CDS platform to other health workers.

## Discussion

This study demonstrated that, when compared with standard practice of CHWs in Kano Nigeria, use of the THINKMD CDS platform led to an observed mean increased adherence to IMCI protocol (a 41.7% change) and improved CHW consistency (a 26% change) in completing each datapoint across individual IMCI evaluations. This is consistent with prior published research, which demonstrated that CDS or digital health is an effective tool for improving CHW’s adherence to clinical protocols in developing countries [16]. The compliance with IMCI guidelines with which CHWs (30.73%) who were part of this study captured data points when using paper booklets in Phase 1 are similar to those documented in a study conducted in Kenya, Uganda, and Tanzania, where fewer than 33% of the health workers assessed all three IMCI danger signs (inability to eat/drink, vomiting, and febrile convulsions) and less than 60% assessed the three main IMCI symptoms of cough/difficult breathing, diarrhea, and fever [17]. In this study, CHWs captured these danger signs and key indicators of dehydration, malnutrition, and respiratory distress more frequently when they used the CDS platform as compared to the standard IMCI paper booklet [10% vs. 83% (skin turgor); 8% vs. 83% (pitting edema; 6% vs 81% (respiratory rate)].

With respect to usability and acceptability, CHWs found the CDS platform easy to use and reported that it improved their ability to conduct IMCI evaluations, and 82% would highly recommend its use to other CHWs (net promoter score = 82%). These findings are consistent with acceptability and usability rates reported by various frontline healthcare workers (FHWs), including CHWs, nurses, and physicians in previous studies on the CDS platform conducted in four different country contexts [15]. Similar to the sentiments shared by CHWs in this study, FHWs in those studies noted that the platform helped them to “do their job better” and with “more confidence”.

Study findings also suggest that consistent and frequent use of the CDS platform helps CHWs to reinforce IMCI knowledge and skills. Countries implementing IMCI report that training, on-the-job supervision, and maintenance of knowledge and skill sets are considerable challenges in ensuring adherence to IMCI protocols and best practices [8]. Poor adherence to protocols may result in situations where healthcare workers are unable to identify the key danger signs and symptoms associated with key clinical conditions that if left untreated can result in premature preventable deaths. Digital clinical decision support tools that can help healthcare workers with protocol adherence and skill set acquisition, as well as support on-the-job training are essential in building frontline health workforces that can provide quality clinical evaluations. Data presented here also suggests that the CDS platform can serve as such an educational and training tool by constantly reinforcing adherence to IMCI protocol and best practices. Moreover, the CDS platform, through its consistent real time capture of data points, supports remote supervision by enabling programs to monitor the quality of the evaluations. These real time data also strengthen surveillance efforts alerting programs to high incidence of specific disease and outbreaks.

In addition to improving quality health care capacity, digital CDS platforms must also be able to financially scale in a sustainable way. Training costs for this study in Kano State, including: a 2-day training (distance learning), the attendance of 30 CHWs ($28/CHW), and trainer’s salary, was estimated to be $2134 USD/CHW. Research has found that distance learning can generate up to 70% cost savings when compared to in-person training [18]. In addition, we can further predict additional cost savings with respect to the need for continuous re-training and hiring of trainers in light that in we captured increased skill acquisition by CDS platform’s embedded training capability [19].

A key limitation of this study is that the data points included in the simplified observers’ checklist were selected based on how easily the data points could be observed. These selection criteria may contribute to learned observation bias. Moreover, this approach may also lead itself to observers bias which may contribute to the variation observed in the CHW achievement of datapoints. Indeed, observers bias is evidenced by the 9.6% difference in rates of adherence documented by the observers compared to the digital platform in Week 4 of Phase 2. The research team implemented a passive observation by trained observers to reduce the Hawthorne effect during the observation.

## Conclusion

Digital clinical decision support platforms are continuing to demonstrate their ability to improve the delivery of healthcare services. Prior research has demonstrated that the THINKMD CDS platform can stand alone as a validated clinical decision support tool, and because of the algorithm’s consideration and integration of WHO-based protocols, is now also demonstrating its ability as a tool that will increase adherence to these WHO IMCI protocols especially as it pertains to assessing for critical conditions such as pneumonia and dehydration and their associated danger signs. There is widespread recognition that increased, sustainable, and consistent IMCI adherence improves overall access to and quality of health service provision and will result in in a possible 28 – 32% decrease in child mortality and morbidity. Scaling up the use of THINKMD CDS platform and other similar CHW focused digital health tools has the potential to significantly increase accurate diagnosis of diseases and effective case management, leading to a reduction in mortality and premature deaths.

### Supplementary Information

Supplementary Material 1.

## Data Availability

The datasets used and/or analyzed during the current study are available from the corresponding author on reasonable request.

## Abbreviations

WHO: World Health Organization
IMCI: Integrated Management of Childhood Illness
U5MR: Under-5 mortality rate
UNICEF: United Nations Children's Fund
LMIC: Low and middle-income countries
CHW: Community health workers
LGA: Local Government Areas
GOPD: General out-patient department
MCH: Maternal and child health
PHC: Primary Health Center
