# Supplementary material for: Adherence to integrated management of childhood illness (IMCI) guidelines by community health workers in Kano State, Nigeria through use of a clinical decision support (CDS) platform
Source: BMC Health Serv Res. 2024 Aug 20;24:953. doi: 10.1186/s12913-024-11245-z (PMC11337650; doi:10.1186/s12913-024-11245-z)
Supplement: Supplementary file 1 — Supplementary Material 1. [file 12913_2024_11245_MOESM1_ESM.pdf]

eHealth AFRICA

INTEGRATED MANAGEMENT FOR CHILDHOOD ILLNESS OBSERVATION CHECKLIST

**Local Government Area:**	**CHW ID:**
**Name of Health Facility:**	**Date:**
**Observer ID:**	**Time:**

**Data Point**	
The CHW asked for the age of the patient	☐
The CHW weighed the patient	☐
The CHW took patient's temperature	☐
The CHW asked for the patient's history of convulsion	☐
The CHW asked if the child had a cough	☐
The CHW asked if patient had a runny nose	☐
The CHW asked if the patient has been vomiting	☐
The CHW asked if the patient was feeding normally	☐
The CHW asked if the patient was stooling more than usual	☐
The CHW asked about blood in stool	☐
The CHW examined the neck by tilting the head forward	☐
The CHW asked the patient to open his/her mouth and looked inside	☐
The CHW examined patient's chest with a stethoscope	☐
The CHW counted the patient's breath per minute by using a watch/cell phone	☐

The CHW pinched the skin of the belly and watched it return to normal shape	☐
The CHW examined patient's skin for rashes by exposing the skin on the arms, legs, chest and back	☐
The CHW pushed on the top of both feet and checked if it went inwards making an indentation	☐
The CHW wrapped a MUAC strip around the patient's upper arm	☐
The CHW pulled down the lower eyelids to examine eyes	☐
The CHW looked into the ear and felt for tender swellings behind the ears.	☐

Overall Assessment

CHW ID:
